# Unravelling TPX2-centered co-expression networks as key drivers of aggressive prostate cancer

**DOI:** 10.1038/s41598-025-27704-4

**Published:** 2025-12-16

**Authors:** Raheleh Sheibani-Tezerji, Carlos Uziel Perez Malla, Gabriel Wasinger, Katarina Misura, Astrid Haase, Anna Malzer, Jessica Kalla, Loan Tran, Gerda Egger

**Affiliations:** 1 Ludwig Boltzmann Institute Applied Diagnostics, Vienna, Austria; 2 Department of Pathology, Medical University of Vienna, Vienna, Austria; 3 Comprehensive Cancer Center, Medical University of Vienna, Vienna, Austria

**Keywords:** Machine learning, WGCNA, RNA-Seq, SHAP, Unsupervised consensus clustering, Prostate cancer, Biomarker

## Abstract

Prostate cancer (PCa) progression is driven by complex molecular reprogramming, yet distinguishing indolent from aggressive disease remains a challenge. We performed an integrative transcriptomic analysis of 1232 PCa samples spanning normal prostate and all major disease stages including primary localized tumors, metastatic hormone-sensitive PCa (mHSPC), and metastatic castration-resistant PCa (mCRPC). By integrating unsupervised consensus clustering (ATC:hclust), weighted gene co-expression network analysis (WGCNA), and explainable machine learning (ML), we identified key transcriptional programs and biomarkers associated with cancer initiation and disease progression. Our analysis revealed persistent dysregulation of mitotic control, DNA damage repair, transcriptional regulation, and cytoskeletal remodeling, underscoring their functional relevance for PCa progression. We uncovered *TPX2* as a central hub gene, consistently upregulated across all disease stages and co-expressed with 21 commonly upregulated genes. ML-based gene ranking and interaction analysis identified connections among the commonly upregulated genes, highlighting *CENPA-MYBL2* for primary localized PCa, *EXO1-NEIL3* for mHSPC and *CENPA-RRM2* for mCRPC. Stage-specific analysis further identified key drivers of distinct disease transitions including *EZH2* and *PLK1* as major regulators of androgen dependence in mHSPC, and *TERT* as a hallmark of mCRPC, highlighting its role in telomere maintenance and tumor progression. This study demonstrates that unsupervised clustering combined with WGCNA and ML enables the discovery of clinically relevant molecular signatures in PCa. Our findings establish *TPX2*-centered networks together with biological pathways implicated in mitotic regulation and DNA damage repair as key drivers of tumor evolution, providing a biologically informed source for biomarker development, drug testing and mechanistic studies.

**Supplementary Information:**

The online version contains supplementary material available at 10.1038/s41598-025-27704-4.

## Introduction

Prostate cancer (PCa) is the second most common cancer in men with 1.41 million cases worldwide in 2020, accounting for an incidence of 23.6 and mortality of 3.4 per 100,000 cases^1^. Risk factors include age, family history, race, germline mutations and clinical predisposition^1^. Clinically, PCa encompasses a spectrum of disease progression, ranging from slow-growing, indolent tumors that can be effectively managed with surgery and/or radiation therapy, to aggressive disease including metastatic hormone-sensitive PCa (mHSPC) and metastatic castration-resistant PCa (mCRPC)^2^. Approximately 20–40% of patients experience biochemical recurrence (BCR) defined by rising prostate specific antigen (PSA) levels after radical prostatectomy due to local recurrence or metastasis. Metastasis most frequently involves bone, distant lymph nodes, liver and lungs and represents the main cause of disease related mortality.

PSA is still the most widely accepted clinical biomarker, which is used for screening, diagnosis, monitoring, and risk prediction of PCa. PSA screening has limitations due to overdiagnosis, leading to unnecessary prostate biopsies and aggressive treatment regardless of the risk^3^. Additionally, prostate-specific membrane antigen (PSMA), a transmembrane protein, is highly overexpressed in PCa (100- to 1000-fold) and is a theranostic target of PCa, both for diagnosis and treatment in nuclear medicine^4^. Expression levels of PSMA are positively correlated with more aggressive disease, high PSA, high Gleason scores and early recurrence.

PCa risk stratification at diagnosis and treatment decisions are currently based on clinical parameters including Gleason score, PSA level and tumor staging. In addition, several risk stratification methods to predict BCR, metastasis or for therapy selection have been tested and validated based on gene expression signatures, some of which are already in clinical use^5–7^. Gene expression analysis including bulk, single cell and spatial RNA sequencing data provided a large array of data and potential biomarkers for primary or metastatic PCa diagnosis or prognosis^5–8^. Moreover, the implementation of advanced computational tools including machine learning approaches has resulted in multiple meta-analyses^9–12^ of publicly available datasets and databases such as The Cancer Genome Atlas (TCGA)^13^. Aside from biomarker discovery, access to public data provides significant insight into understanding biological pathways, modifications, and functions of established biomarkers in patients. In silico data mining is a useful tool to decipher relevant disease biomarkers and to generate research hypotheses. In particular, various machine (deep) learning algorithms have been developed and also applied to PCa data, which have high potential to interrogate large amounts of multi-dimensional data^14–18^. Most studies so far, were directed towards specific tumor stages, to infer markers for primary PCa, mHSPC or mCRPC, or towards therapeutic responses, to facilitate patient stratification and therapy selection^19–27^.

Rather than using group-wise comparisons, the current study integrated publicly available data from benign prostate, primary PCa, mHSPC and mCRPC to identify common genes and pathways driving PCa development and progression towards androgen-independent metastatic disease. We first assessed major transcriptional changes across all sample types using likelihood ratio testing (LRT) and unsupervised hierarchical clustering, followed by identification of co-expression gene networks by weighted correlation network analysis (WGCNA). Importantly, we assessed differentially expressed genes (DEGs) on pathway level and identified *TPX2* as a central hub gene in gene clusters comparing normal to primary PCa, primary PCa to mHSPC, and mHSPC to mCRPC. These clusters contained genes important for mitosis, cell cycle and transcriptional regulation, and shared 22 differentially expressed genes, which were significantly associated with worse prognosis. Lastly, we employed machine learning to rank the predictive nature of the 22 genes for individual disease stages and identified three gene pairs specific for detecting primary localized, hormone sensitive or castration resistant metastatic disease. We suggest that these genes represent important biological drivers of PCa progression and might prove useful as diagnostic and prognostic markers as well as therapeutic targets in the future.

## Results

**Fig. 1:**
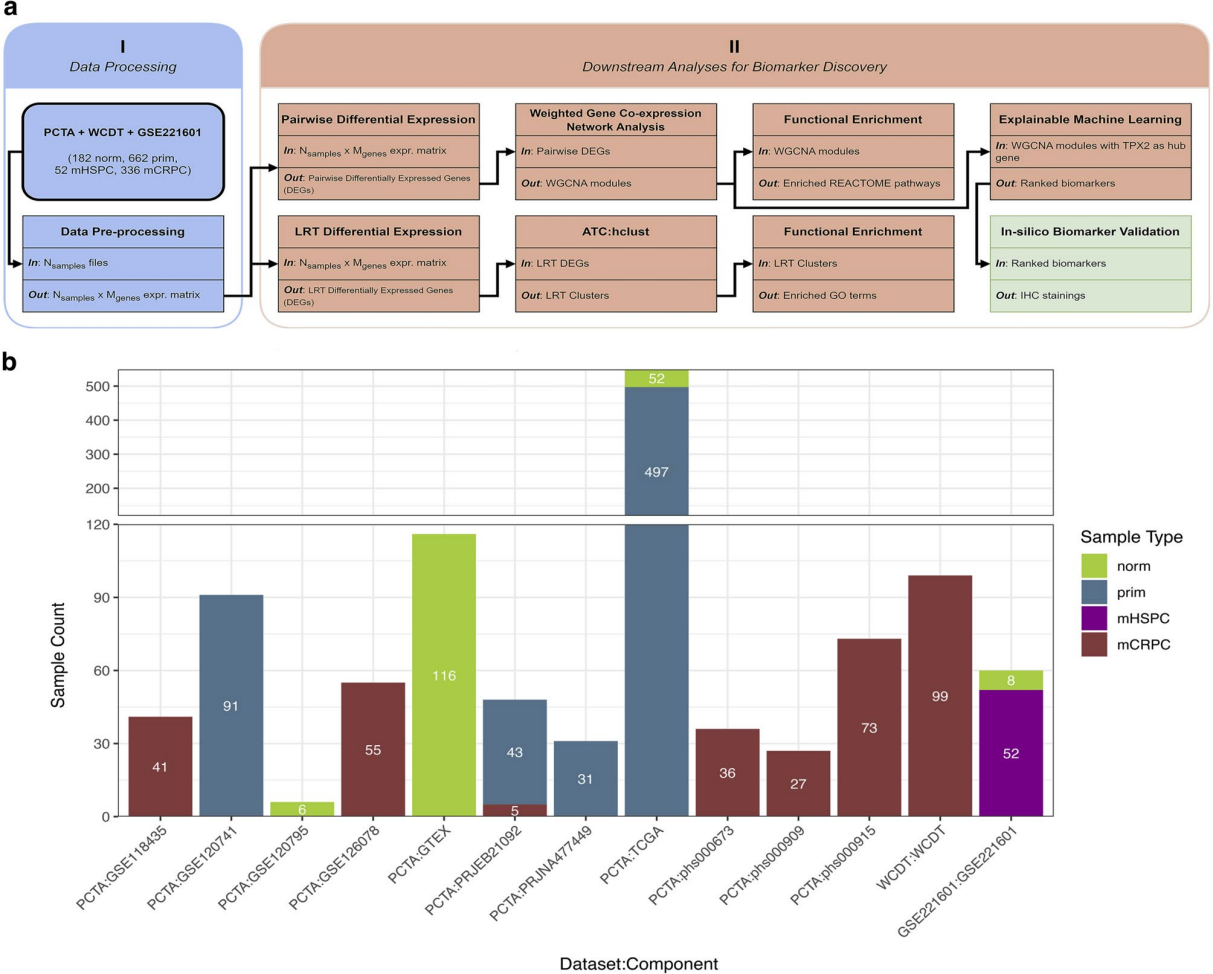
Analysis setup. (**a**) Schematic presentation of the analysis workflow. The diagram shows the 4 main analysis steps: data processing, differential analyses (pairwise, LRT), WGCNA and ML analysis. (**b**) Overview of individual datasets, including number of samples and sample type distribution. A total of 182 normal (norm), 662 primary localized PCa (prim), 52 mHSPC and 336 mCRPC samples were included in this analysis.

To infer PCa-specific gene expression signatures that reflected general transcriptional trajectories of PCa development and progression irrespective of molecular subtype, we conducted a comprehensive transcriptomic analysis of 1232 PCa samples (see analysis workflow in Fig. 1a). Our study utilized three major datasets: the PCa Transcriptome Atlas (PCTA)^23^, the West Coast PCa Dream Team mCRPC (WCDT-MCRPC)^28,29^ and GSE221601 (sourced from the Gene Expression Omnibus, (GEO)^30,31^. PCTA comprises a curated collection of 11 datasets, featuring RNA-Seq samples spanning normal, primary, and mCRPC tissues. Among the largest contributors to PCTA are TCGA-PRAD^13^, GTEx^32^, GSE120741^33^, and phs000915^34^. Additionally, mHSPC samples were sourced from GSE221601. In total, our study included 182 normal (norm), 662 primary localized tumor (prim), 52 mHSPC, and 336 mCRPC samples (Fig. 1b and Supplementary Table S1).

### Global scaling of gene expression profiles unveils (dis)similarities between normal and tumor samples

**Fig. 2:**
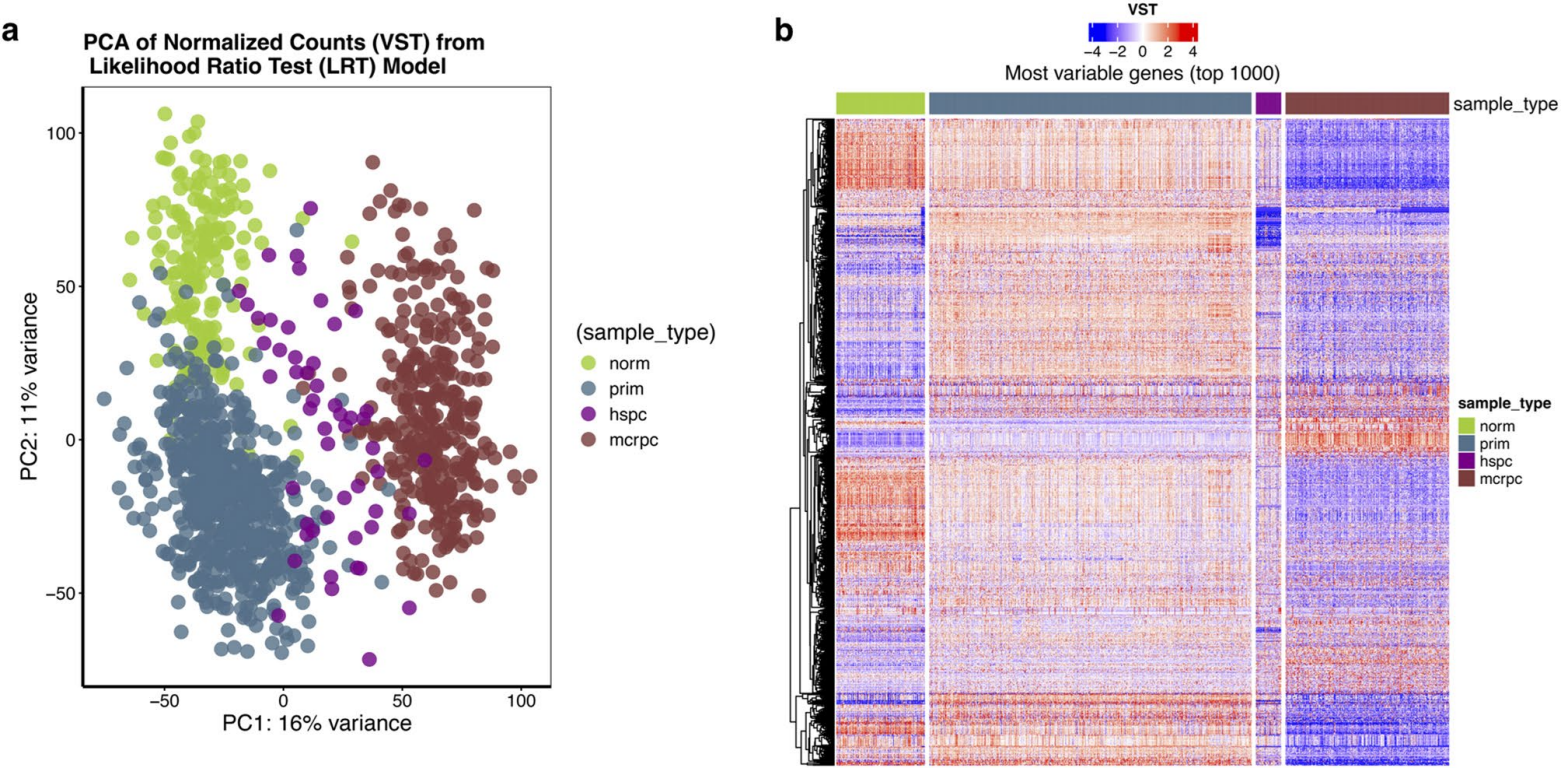
Global scaling of RNA-Seq data. (**a**) Principal component analysis (PCA) of RNA expression levels. The first two components shown explain the largest part of the variation in RNA expression (37%). Individual samples (circles) are color-coded by sample type (norm, prim, mHSPC, mCRPC). (**b**) Heatmap of the unsupervised hierarchical clustering analysis on the variance-stabilized transformed (VST) expression of the top 1000 most variable genes across all samples within each sample type. Each row corresponds to a single gene, whereas each column corresponds to a single sample.

In order to assess the similarity between different sample types based on their gene expression signatures, we performed principal component analysis (PCA) (Fig. 2a). PCA revealed that the majority of the variation in expression levels was attributable to differences among sample types. A clear separation was observed for mCRPC samples from mHSPC, prim, and norm samples, with some overlap between norm and prim tissues. These differences accounted for approximately 27% of the total variance, distributed across the two major principal components (PC1 = 16%, PC2 = 11%).

Unsupervised hierarchical clustering of normalized gene expression data (VST) using the top 1000 most variable genes revealed a striking pattern of expression changes across PCa progression (Fig. 2b). A subset of highly expressed genes in norm samples showed gradual changes in expression levels in prim and mHSPC samples, ultimately displaying very low expression in mCRPC samples. Conversely, other clusters of genes showed the opposite trend, with low expression in normal samples and gradually increasing expression in primary tumors and mHSPC before reaching their highest expression levels in mCRPC. These gradual shifts in gene expression highlight the progressive reprogramming during PCa progression, distinguishing normal and malignant prostate tissues, while emphasizing transcriptional differences between localized and advanced disease stages.

### Unsupervised clustering reveals robust differentially expressed gene signatures for PCa

We initially applied the DESeq2 likelihood ratio test (LRT) to assess the overall effect of sample types on PCa progression. This approach allowed us to identify global transcriptional changes across all conditions without restricting the analysis to specific pairwise contrasts. By leveraging the full dataset, we ensured that our DEGs and pathway enrichment analysis captured broad biological themes relevant to disease progression rather than being limited to individual transitions. We focused exclusively on DEGs with unique gene IDs (i.e., ENTREZID) and, unless otherwise specified, filtered by Padj < 0.05 and absolute LFC > 1. The differential expression analysis identified 5106 DEGs. Among these, 2895 genes were upregulated, while 2211 were downregulated (Supplementary Table S2), reflecting widespread molecular alterations associated with disease progression. The large-scale transcriptional reprogramming captured in this analysis provides a high-resolution molecular framework for biomarker discovery and therapeutic target identification in advanced PCa.

To define high-confidence gene expression signatures associated with PCa progression, we applied unsupervised hierarchical clustering to the DEGs from the LRT analysis. This data-driven approach enabled a more accurate and biologically meaningful identification of transcriptional signatures. We employed the Ability to Correlate to other rows (ATC) method implemented in the cola R package^35^. ATC is specifically designed as a feature selection strategy for consensus partitioning and can be combined with various clustering algorithms, including hierarchical clustering (hclust)^35^. We applied this combined method, ATC:hclust, to the set of 5,106 DEGs.

**Fig. 3:**
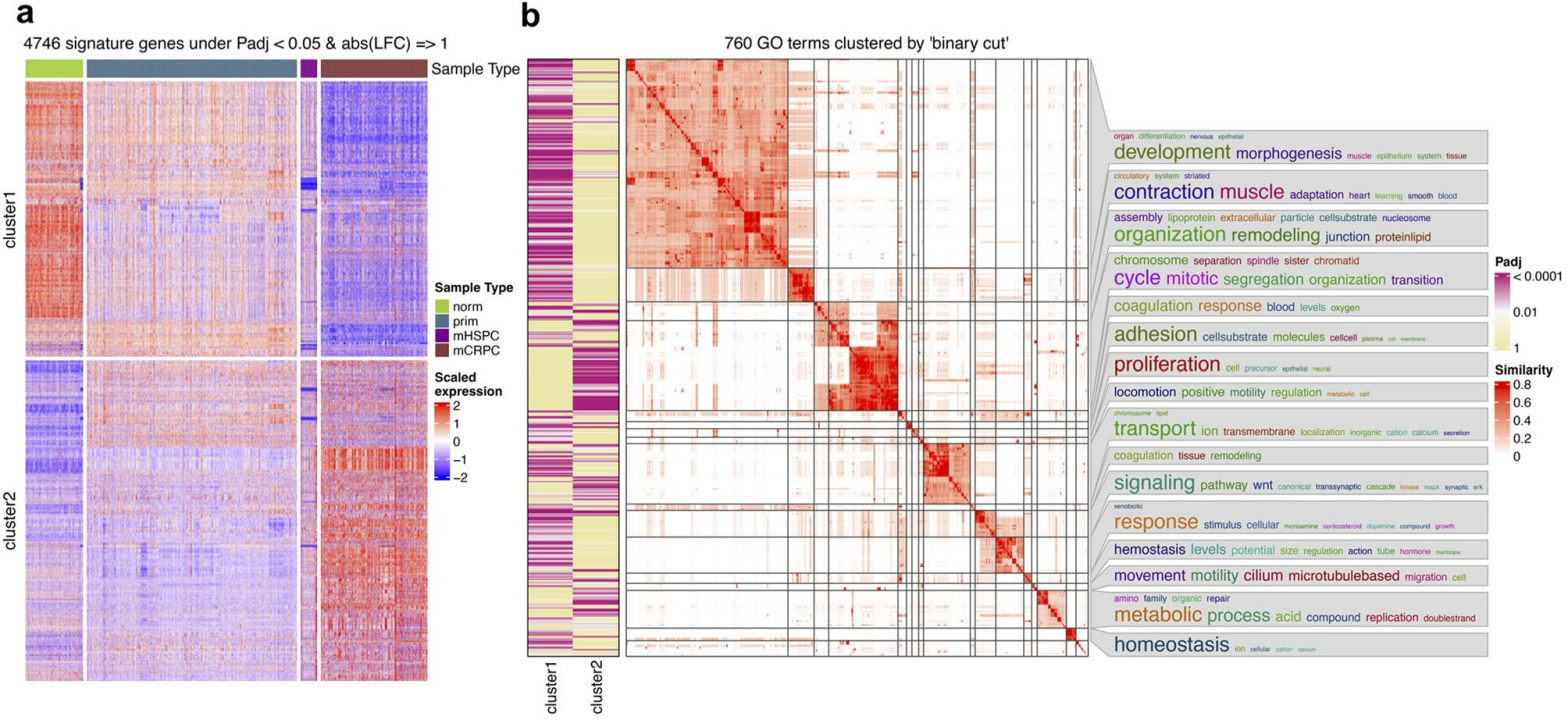
Expression patterns and biological function of differentially expressed genes. (**a**) Hierarchical clustering of signature genes differentially expressed in the progression from norm to mCRPC. Rows correspond to genes and columns correspond to samples. Sample class was obtained by consensus partitioning methods to separate samples into subclasses. The two gene clusters (cluster1 and cluster2) were generated by applying k-means clustering on rows of the expression matrix. The z-score standardization was applied on matrix rows. (**b**) Significant GO terms enriched in either of the corresponding gene clusters were grouped based on their similarities. The columns on the left of the GO heatmap indicate the significance levels of GO terms in the individual clusters (Padj < 0.05). The summaries of the biological functions in clusters are visualized as word clouds and are attached to the GO similarity heatmap. In word clouds, enrichment of keywords is assessed by Fisher’s exact test, and the significance is mapped to the font size of keywords.

We excluded low-variance genes (sd ≤ 0.05 quantile, n = 360 DEGs removed), resulting in 4746 robust signature genes across all PCa stages (Fig. 3a). The classification of samples using consensus partitioning revealed distinct clusters, with mHSPC and mCRPC samples clearly separated, indicating transcriptomic signatures linked to specific disease stages. In our analysis, ATC:hclust facilitated the stratification of DEGs into two distinct expression clusters (up- and downregulated), highlighting biologically cohesive gene signatures associated with different stages of PCa progression.

To further characterize the functional relevance of these gene clusters, we applied *simplifyEnrichment* for functional classification of enriched Gene Ontology (GO) terms^36^ and their associated DEGs (Fig. 3b and Supplementary Table S3). This method preserved the biological specificity of pathway associations by grouping functionally similar terms into contextually meaningful clusters. Among the most significantly enriched pathways, cell cycle regulation and chromosomal dynamics were highly represented, including processes such as chromosome segregation, mitotic spindle organization, and sister chromatid separation, highlighting the role of genomic instability during progressive disease. Cell adhesion and extracellular matrix remodeling pathways were also enriched, particularly in relation to cell-substrate interactions and epithelial tissue remodeling, suggesting their involvement in tumor invasion and metastatic potential. Additionally, Wnt signaling, MAPK signaling, and synaptic signaling cascades were among the top enriched pathways, reflecting the importance of transcriptional regulation, kinase activity, and intercellular communication in PCa progression. Metabolic reprogramming, particularly related to lipid metabolism, calcium homeostasis, and amino acid transport was also observed, further emphasizing the metabolic adaptations in aggressive prostate cancer phenotypes.

Together, these transcriptional programs show conserved molecular alterations that drive prostate cancer progression and provide mechanistic insight into the aggressive phenotypes observed in advanced disease stages.

### Cell surface protein analysis reveals key biomarkers involved in tumor progression and microenvironment interactions

**Fig. 4:**
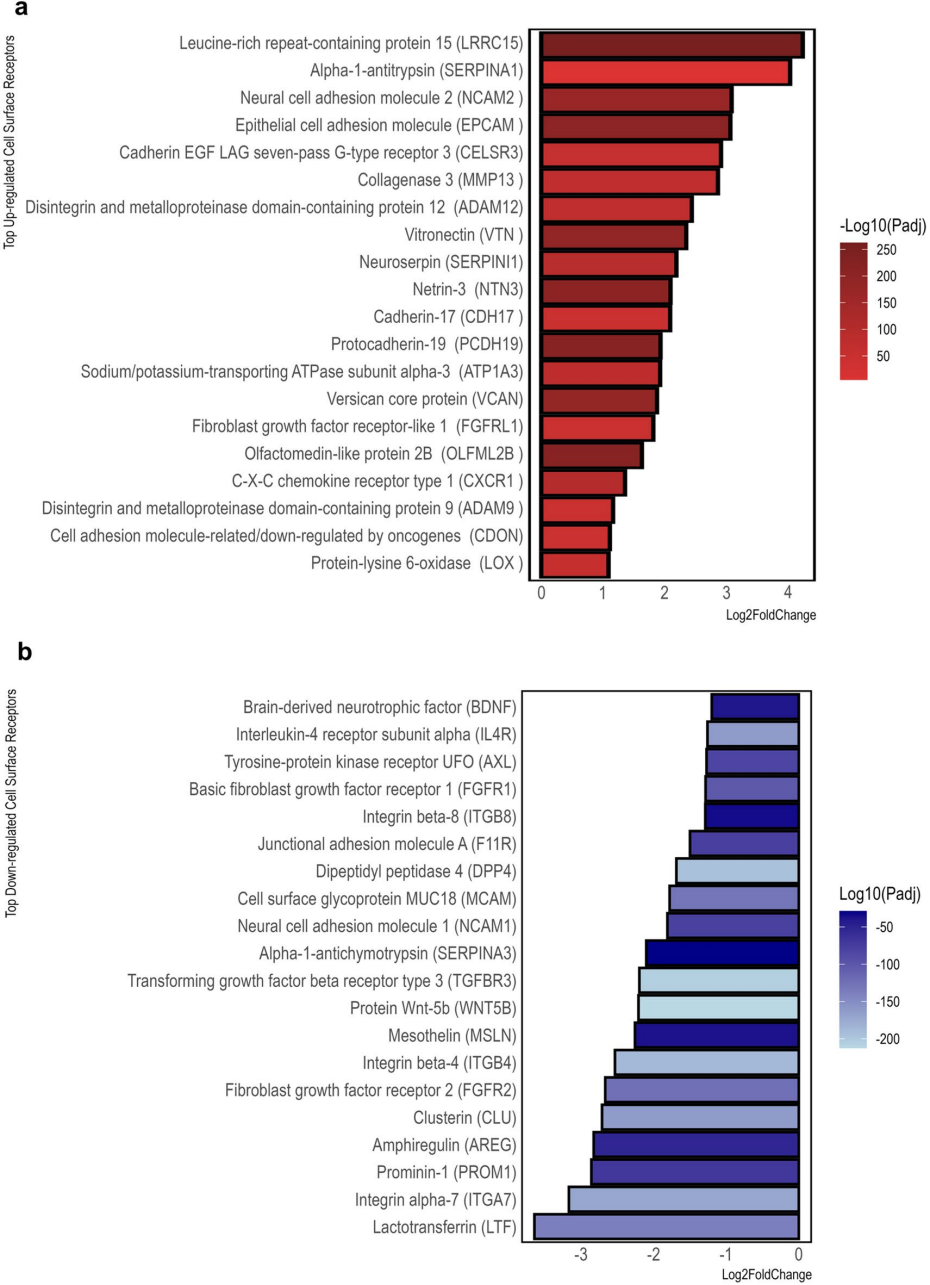
Mining of cell surface protein receptors. Cell surface proteins identified among the significantly (**a**) upregulated and (**b**) downregulated DEGs in the progression from norm to mCRPC. Adjusted *P* values are represented by colors and log2fold changes are represented by bar length.

Cell surface proteins such as receptors or transporters represent suitable targets for specific diagnostic and therapeutic applications such as molecular imaging or targeted therapies. Therefore, we utilized the Cell Surface Protein Atlas (CSPA)^37^ in order to identify upregulated cell surface proteins (CSPs) among the DEGs identified by LRT as described above. The CSP analysis resulted in a total of 322 proteins, of which 159 were significantly upregulated and 163 were significantly downregulated across all sample types (Supplementary Table S4). The upregulation of genes encoding matrix associated factors including proteases and cell adhesion molecules such as *ADAM12*, *NTN3*, *LRRC15*, *CDH17*, *PCDH19*, *VTN* highlighted the relevance of the tumor microenvironment and cell–cell and cell–matrix interaction for progression to metastatic disease (Fig. 4a). Interestingly, several of the identified genes are related to neuronal development or function including *NTN3*, and *SERPINI1*.Meanwhile, downregulated genes encoding for cell surface proteins included growth factors and receptors implicated in prostate development or homoeostasis (e.g. *BDNF*, *FGFR2*), as well as cell adhesion molecules (e.g. *ITGB4*, *ITGA7*, *NCAM1*) suggesting a disruption of normal cell attachment, cell–cell interactions and communication mechanisms in tumor cells (Fig. 4b).

### Weighted gene co-expression network analysis reveals *TPX2* as a central marker of PCa progression

While the LRT approach is well-suited for identifying global transcriptional patterns, it does not directly provide genes that best distinguish specific conditions and transitions between sample types. Weighted Gene Co-expression Network Analysis (WGCNA)^38^, which identifies clusters (modules) of highly correlated genes across samples, requires well-defined binary comparisons to learn discriminatory features. Therefore, we applied pairwise differential gene expression analysis for each transition from prim/norm, mHSPC/prim and mCRPC/mHSPC (Supplementary Table S5). Subsequent WGCNA analysis was performed separately for up- and downregulated DEGs (Padj < 0.05, abs(LFC) > 1), identifying modules for each group comparison (Supplementary Table S6).

**Fig. 5:**
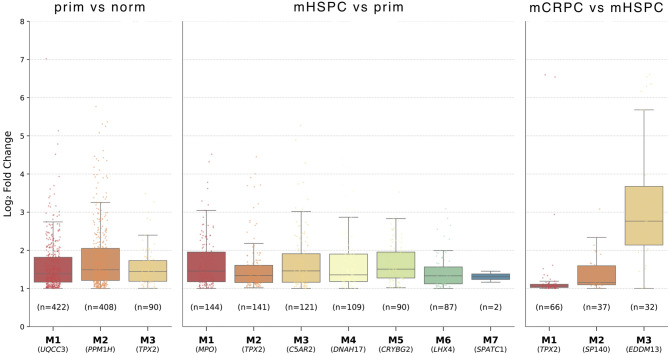
Weighted gene co-expression network analysis. Distribution of Log2 fold changes of the up-regulated DEGs in the WGCNA modules identified in prim/norm, mHSPC/prim and mCRPC/mHSPC comparisons. The total number of DEGs per module, as well as their hub gene are highlighted.

For downregulated DEGs, one module was detected for each prim/norm and mCRPC/mHSPC, while nine modules were identified for mHSPC/prim group comparisons. (Supplementary Table S6). Notably, the mHSCP/prim clusters contained mainly non-coding RNA genes, which might reflect residual technical biases and differences in sequencing protocols, despite extensive harmonization efforts across datasets. Specifically, mHSPC samples were sequenced using QuantSeq 3’ libraries, in contrast to the poly-A enriched protocols used for PCTA and WCDT samples, which will also produce data for non-coding RNA species lacking polyA tails. Focusing further on clusters identified from upregulated DEGs, we identified three modules in both the prim/norm and mCRPC/mHSPC comparisons, while the mHSPC/prim comparison yielded seven modules, potentially reflecting broad transcriptional reprogramming as tumors transition from localized to metastatic androgen-sensitive states (Fig. 5).

**Fig. 6:**
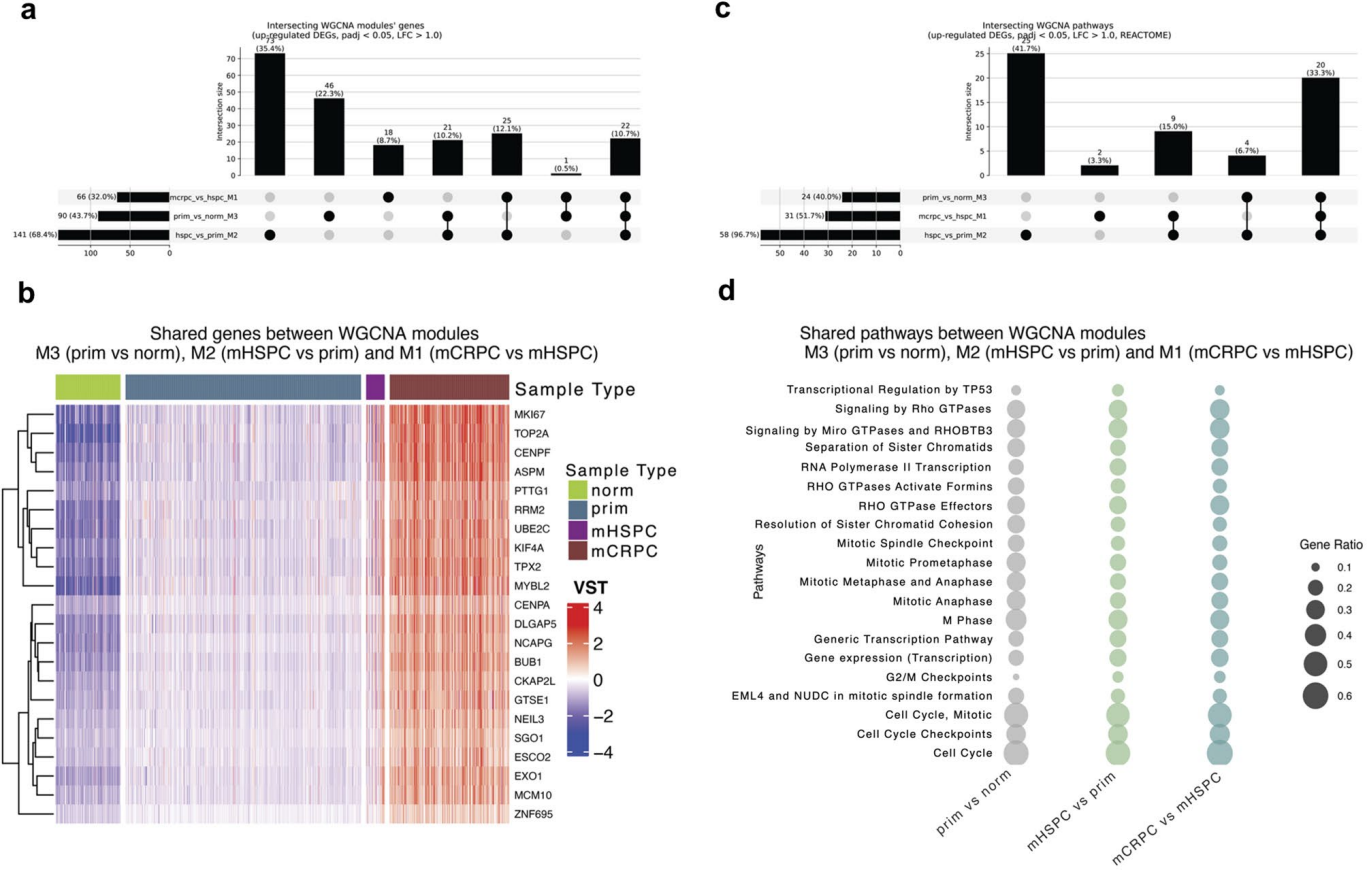
Shared genes and pathways between *TPX2* WGCNA modules. (**a**) Upset plot showing shared DEGs between WGCNA modules related to *TPX2* as a hub gene, including M3 (prim/norm), M2 (mHSPC/prim) and M1 (mCRPC/mHSPC). A total of 22 DEGs were shared among all three modules. (**b**) Heatmap of the unsupervised hierarchical clustering analysis on the variance-stabilized transformed (VST) expression of shared DEGs among all *TPX2* modules as in (a). Each row corresponds to a single gene, whereas each column corresponds to a single sample. (**c**) Upset plot showing shared REACTOME pathways between modules as in (a). (**d**) A total of 20 pathways were shared among all three modules as in (a). The circle size is the gene ratio for each shared pathway in each comparison.

*TPX2*, a microtubule associated protein essential for mitotic spindle assembly, was identified as a hub gene in all three group comparisons, highlighting its pivotal role in PCa progression. The *TPX2* clusters contained 90, 141 and 64 significantly upregulated genes in the prim/norm, mHSPC/prim and mCRPC/mHSPC comparisons, respectively, and 22 genes (including *TPX2*) were shared among all three clusters (Fig. 6a). Unsupervised hierarchical clustering of the 22 genes showed a gradual increase of their expression from normal prostate to mCRPC and allowed for separation of the individual disease stages (Fig. 6b). Interestingly, 15 out of the 22 shared genes were identified as hub genes in PCa in previous studies^39–43^.

To gain a broader understanding of *TPX2*-associated networks, we performed functional enrichment analysis of DEGs within *TPX2*-centered clusters for each comparison using the REACTOME database (Supplementary Table S7).We identified 20 shared REACTOME pathways among modules M3 (prim/norm), M2 (mHSPC/prim) and M1 (mCRPC/mHSPC), which were mainly associated with mitosis, cell cycle and transcriptional regulation (Fig. 6c, d) highlighting these essential molecular mechanisms to be consistently deregulated across all disease stages, reinforcing their central role for PCa progression. Key pathways such as M phase, mitotic anaphase, separation of sister chromatids, and G2/M checkpoints indicate that PCa cells exploit *TPX2*, a hub gene across all modules, and an essential gene for mitotic spindle assembly and Aurora kinase A (AURKA) activation^44^ (Fig. 6d and Supplementary Fig. S1). Enriched pathways, including transcriptional regulation by TP53 and RNA polymerase II transcription, suggest that PCa cells fine-tune transcription to sustain rapid proliferation^45^. Alterations in Rho GTPase signaling play a significant role in PCa progression by driving cytoskeletal remodeling, which enhances cell migration and invasion, thereby linking chromosomal instability to metastasis^46^.

Lastly, to determine the clinical relevance of the 22 shared genes, we performed recursive partitioning (RP) based survival analysis^47^ employing the prostateCancerTaylor R package^48^, and univariate Cox proportional hazards analyses using the Memorial Sloan Kettering Cancer Centre (MSKCC) database^49^, which includes primary and metastatic PCa samples. These analyses revealed that 20 out of the 22 genes (there was no information available on this database for the gene *SGO1*) were significantly associated with shorter time to and increased risk of BCR (Supplementary Fig. S2). Thus, these 22 genes might represent a valuable prognostic signature, which should be evaluated for their clinical applicability in future clinical cohorts. Of note, several of the 22 genes are also part of already established diagnostic tests including Prolaris^50^ (*ASPM*, *CENPF*, *DLGAP5*, *MCM10*, *PTTG1*, *RRM2*, *TOP2A*), Decipher^51^ (*UBE2C*) and Oncotype DX^52^ (*TPX2*).

Next, the genes that were specific to each of the above *TPX2* modules for each transition were identified. We identified a total of 46 genes only expressed in module M3 (prim/norm) that play significant roles in PCa progression (Supplementary Fig. S3a). Among these genes, we found *MMP9* (Matrix Metallopeptidase 9), which is involved in the degradation of the extracellular matrix, facilitating tumor invasion and metastasis. Elevated levels of *MMP9* have been associated with increased aggressiveness in PCa^53^. *CCNB1* (Cyclin B1) is another unique gene to this module that is essential for the control of the cell cycle at the G2/M transition. Overexpression of *CCNB1* has been linked to increased cell proliferation and tumor growth in PCa^54^. Notably, of the 73 genes only present in M2 of mHSPC/prim (Supplementary Fig. S3b), *PLK1* and *EZH2* have been identified as significant contributors to PCa progression. *PLK1* (Polo-like kinase 1) is overexpressed in PCa and is associated with higher tumor grades, suggesting its involvement in tumorigenesis and disease progression^55^. Inhibitors targeting *PLK1* have been explored as potential treatments for advanced PCa, aiming to disrupt mitotic processes essential for cancer cell proliferation^56^. *EZH2* (Enhancer of zeste homolog 2) overexpression has been linked to the progression of PCa, particularly in the development of castration-resistant forms of the disease^57^. Targeting *EZH2* with specific inhibitors is being investigated as a strategy to manage advanced PCa, given its role in promoting tumor growth and resistance to conventional therapies^58^. These findings highlight the importance of *PLK1* and *EZH2* in PCa progression and underscore their potential as therapeutic targets. Furthermore, 18 genes specific to module M1 of mCRPC/mHSPC play significant roles in PCa progression (Supplementary Fig. S3c). Genes such as *TERT* (Telomerase Reverse Transcriptase), which is the catalytic subunit of telomerase, an enzyme responsible for maintaining telomere length. Overexpression of *TERT* has been observed in PCa, contributing to cellular immortality and tumor progression. It is reported that consistent overexpression of *TERC* (the RNA component of telomerase) in PCa, driven by *MYC*, suggests a role in tumor progression^59^.

### Validation of *TPX2* as a potential target in PCa

**Fig. 7:**
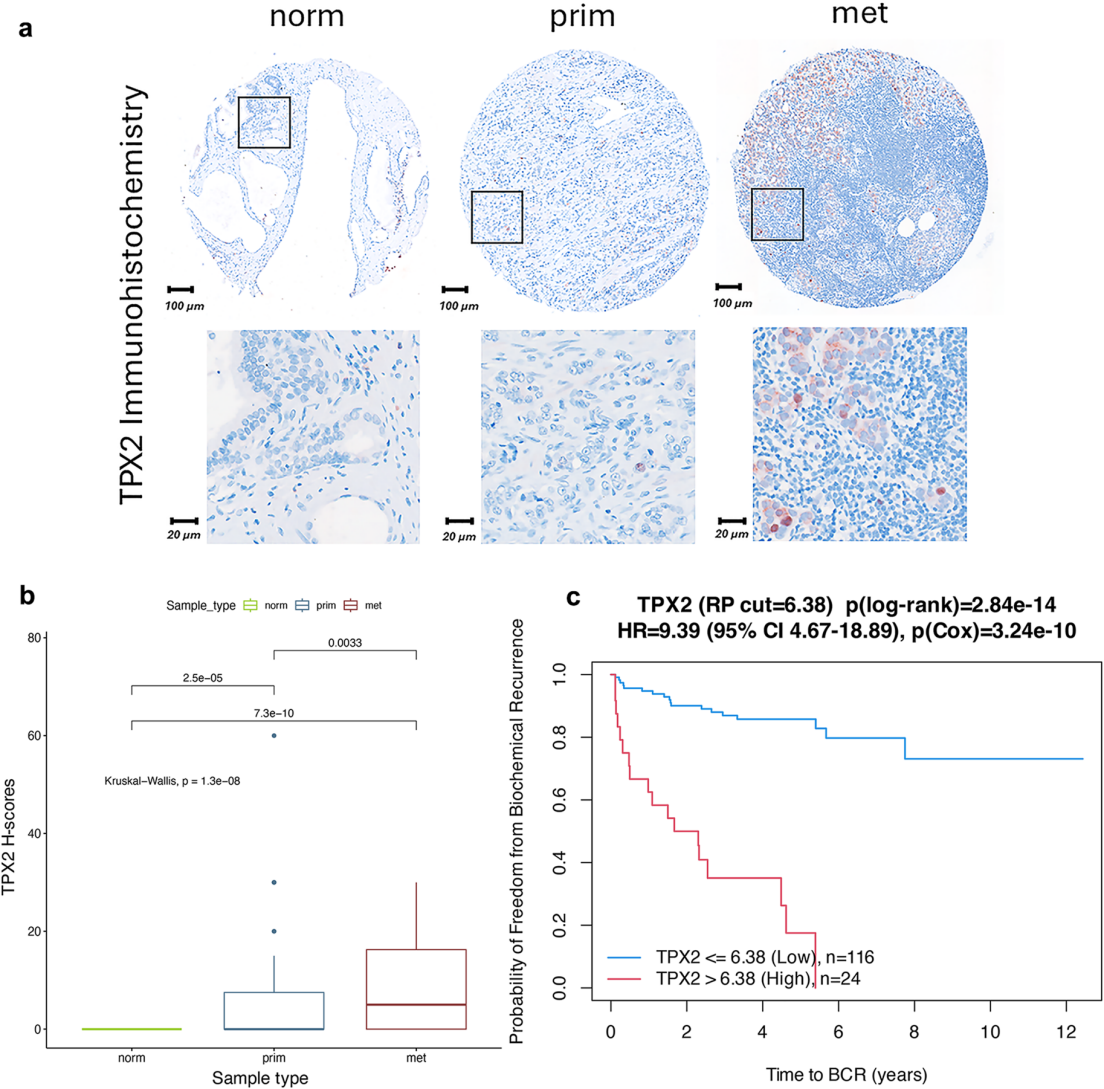
Validation of *TPX2* protein expression. (**a**) Representative microscopic images of IHC staining of normal (norm), primary PCa (prim) and lymph node metastatic (met) PCa samples. Brown nuclear staining indicates *TPX2* expression. Sections were counterstained with hematoxilin (blue color). (**b**) Protein expression levels indicated by H-scores, calculated from tissue microarrays containing 154 norm, 194 prim and 54 met tissue core biopsies. (**c**) Kaplan Meyer survival curves for high (red) and low (blue) expression levels of *TPX2*. The expression cutoff (6.38) was determined by recursive partitioning. Hazard ratio (HR) including 95% confidence interval (CI) were determined by Cox proportional hazards analysis.

As *TPX2* was identified as a hub gene among all group comparisons in WGCNA highlighting its central role for tumor progression, we examined the protein expression levels of *TPX2* on a tissue microarray (TMA) containing 51 treatment naïve primary PCa samples with adjacent normal tissues and 35 matched lymph node metastases. Notably, while normal adjacent prostate epithelia were negative for *TPX2* expression, a gradual significant increased expression was detected in primary PCa and lymph node metastases (Fig. 7a, b). Together, these data confirm the upregulation of *TPX2* during PCa progression on protein level. Moreover, high *TPX2* expression is significantly associated with shorter time to BCR as indicated by survival analysis of primary and metastatic PCa of the MSKCC dataset^49^ (Fig. 7c), implying its biological role for tumor progression and its potential as a biomarker and therapeutic target for advanced PCa. Thus, *TPX2* might have a high potential as an individual marker on protein level, or on RNA level in combination with other co-expressed genes, as already applied by the Oncotype DX test, or within our identified marker panel of 22 progressively upregulated genes.

### Ranking shared genes in *TPX2*-centered modules using explainable machine learning

To refine and weigh our collection of potential biomarkers for PCa progression, we applied machine learning (ML), made explainable via shapely additive explanations (SHAP) values, to rank the 22 shared genes of the three *TPX2* modules previously identified by WGCNA. More concretely, we trained binary classifiers on each pairwise comparison, where the control sample type (e.g. norm) was the negative class and the test sample type (e.g. prim) was the positive class^60^. We then calculated the average first and second order (i.e. pairwise feature interactions) SHAP values for each gene and sample over 8192 training iterations (Supplementary Table S8). We obtained high classification performance for prim/norm and mCRPC/mHSPC, but mHSPC/prim was more difficult to model most likely due to the significant difference in sample sizes (Table 1).

**Table 1:** Machine learning performance metrics.

	Precision	Recall	F1 score	Balanced accuracy
prim/norm	0.960 ± 0.014	0.993 ± 0.008	0.976 ± 0.008	0.920 ± 0.029
mHSPC/prim	0.712 ± 0.202	0.373 ± 0.148	0.471 ± 0.149	0.680 ± 0.073
mCRPC/mHSPC	0.912 ± 0.017	0.977 ± 0.018	0.943 ± 0.013	0.665 ± 0.068

Average (± standard deviation) of precision (probability of a positive prediction being correct), recall (probability of a positive case being detected), F1 score (harmonic mean of precision and recall) and balanced accuracy (average of recall and specificity, probability a negative case being correctly identified) over 8192 training iterations. All metrics are high enough to trust the resulting SHAP values. Balanced accuracy suffers on comparisons including the mHSPC group due to heavy class imbalances.

**Fig. 8:**
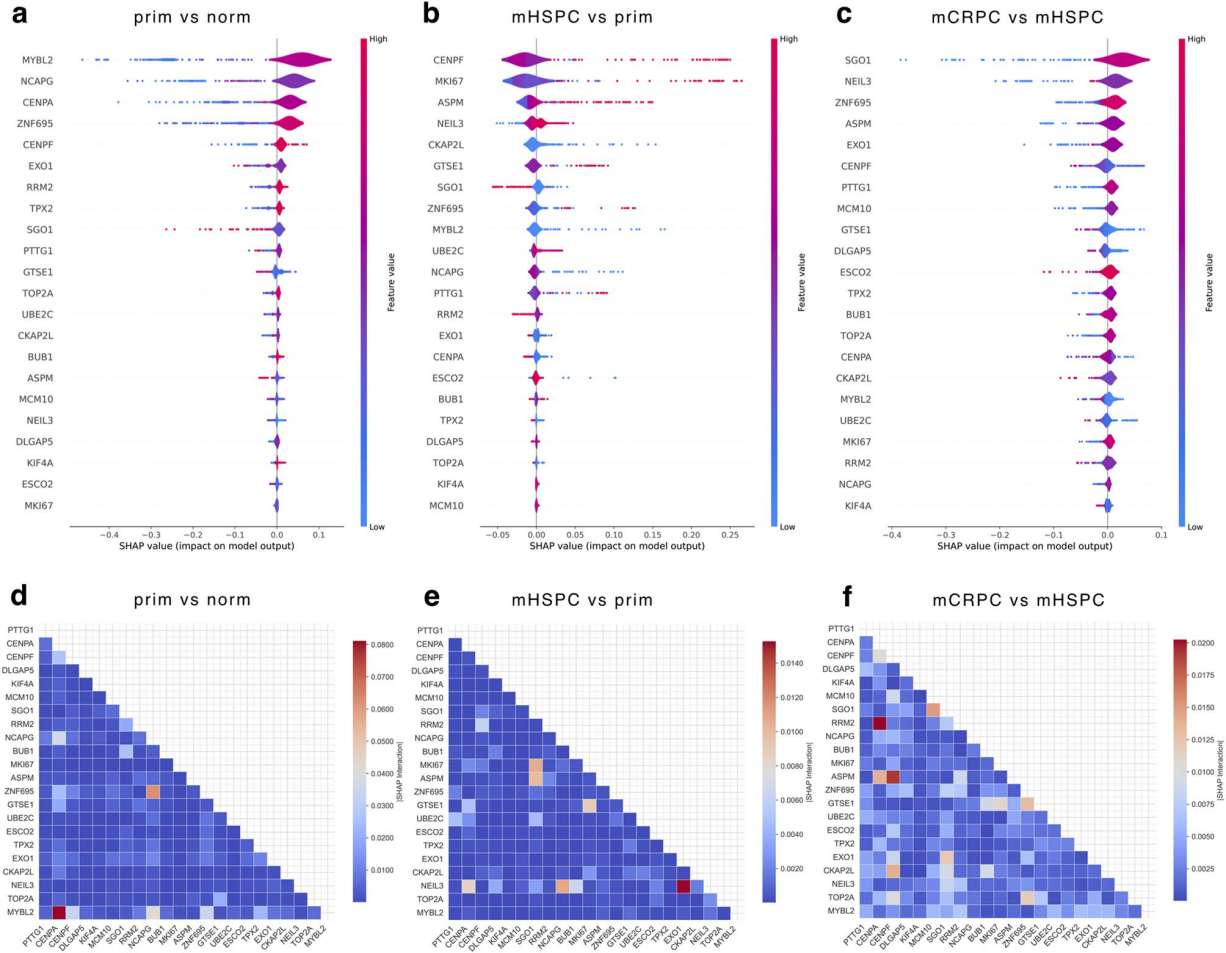
Explainable machine learning. Distribution of bootstrapped SHAP values of the 22 shared DEGs between (**a**) M3 (prim/norm), (**b**) M2 (mHSPC/prim) and (**c**) M1 (mCRPC/mHSPC) obtained from binary machine learning classifiers. The distribution is color-coded by VST gene expression values (from blue, low expression, to red, high expression). The further out a dot is from the separating vertical line, the higher the average SHAP value across bootstrapping iterations, as indicated in the X axis, and hence the higher the impact on the model output. (**d**–**f**) Second-order SHAP values representing feature interaction predictive strength among each of the 22 shared DEGs between M3 (prim/norm), M2 (mHSPC/prim) and M1 (mCRPC/mHSPC) obtained from binary machine learning classifiers.

This approach allowed us to quantify the impact of each gene on sample classification, while maintaining a biological perspective from co-expression networks (Fig. 8a–c). Negative and positive SHAP values steer the model towards a classification output of 0 or 1, representing the two sample groups in the comparison, where 0 represents the control (e.g. norm in prim/norm) and the 1 is the sample type used for the test (e.g. prim in prim/norm). Amongst the most important features, we identified *MYBL2* and *NCAPG* as the strongest predictors for prim, *CENPF* and *MKI67* for mHSPC and *SG01* and *NEIL3* for mCRPC. We then focused on feature interactions (i.e. second-order SHAP values), to account for potential interactions or co-regulation of genes within the 22 candidate genes. Strong pairwise interactions involving *CENPA*-*MYBL2,* and *CENPA*-*RRM2* were identified in the prim/norm and mCRPC/mHSPC transitions (Fig. 8d, f and Supplementary Fig. S4), highlighting their predictive association and providing insights into potential functional dependencies of these genes during tumor initiation and castration resistance. In the intermediate state (mHSPC/prim), *EXO1*-*NEIL3* showed the greatest association (Fig. 8e and Supplementary Fig. S4), possibly reflecting a distinct regulatory shift during progression to hormone-sensitive metastasis.

### Correlation of *CENPA, MYBL2, RRM2, EXO1, NEIL3 and TPX2* with clinical data for PCa progression

**Fig. 9:**
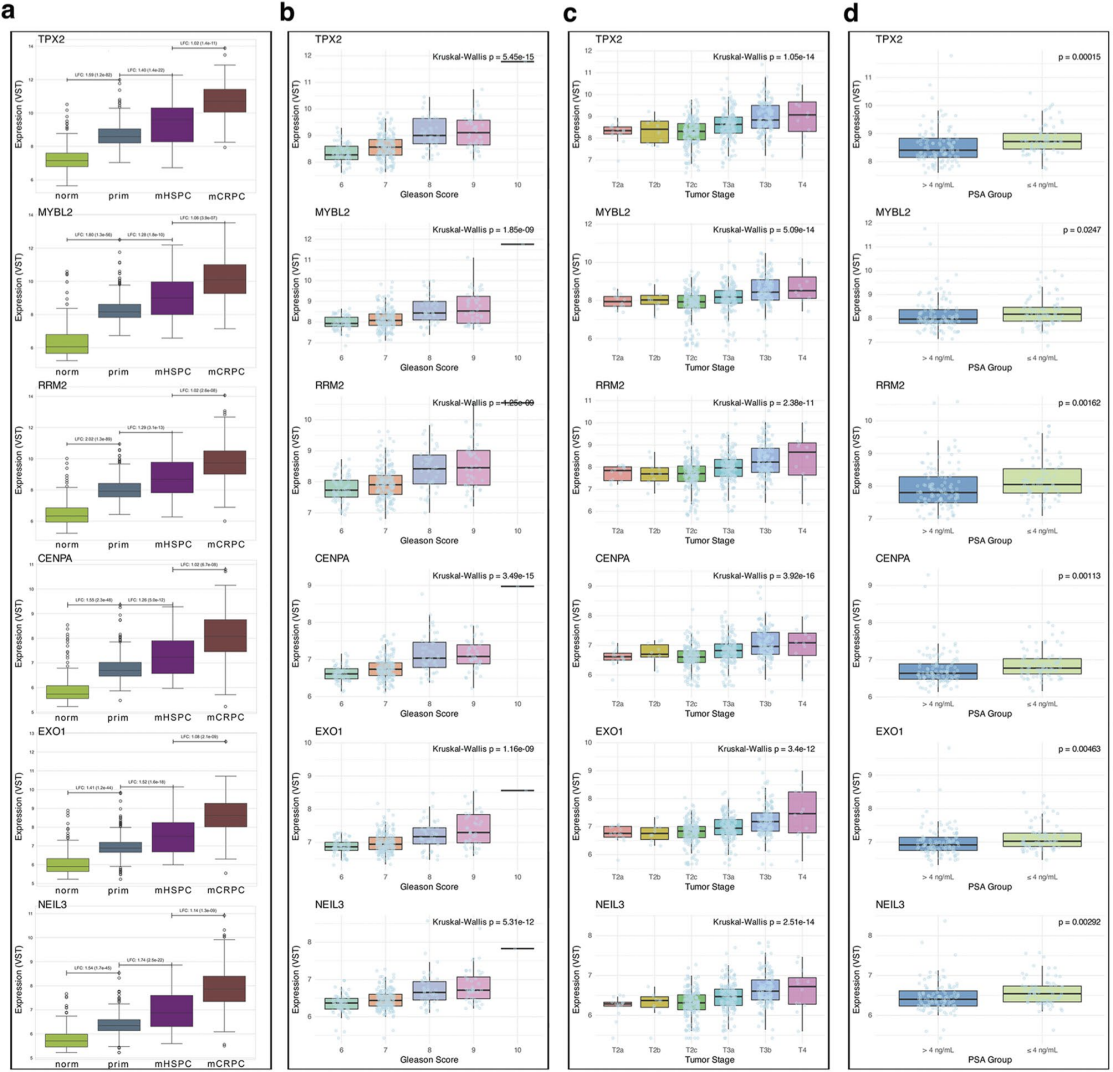
Gene expression of candidate biomarkers associated with clinical data. (**a**) Distribution of VST expression values of candidate biomarkers across sample types, including Log2 fold changes and their corresponding *P* value obtained from DESeq2 differential expression analysis. Distribution of VST expression levels according to (**b**) Gleason score categories, (**c**) Tumor stages, and (**d**) serum PSA levels (PSA ≤ 4 vs. > 4 ng/mL). Statistical significance in panels (b, c) is based on Kruskal Wallis test, while comparisons in panel (**d**) used the Wilcoxon rank-sum test.

Next, we investigated the clinical significance of *TPX2-*associated genes, including *RRM2*, *CENPA, MYBL2, EXO1* and *NEIL3*, which were identified by WGCNA and top ranked through ML analysis, by assessing their prognostic significance in relation to Gleason score, tumor stage, and PSA levels obtained from clinical data from TCGA-PRAD^13^. All six genes were significantly upregulated in the combined dataset during the progression from the normal state to mCRPC (Fig. 9a), TCGA-PRAD data highlighted a significant upregulation of all genes according to increasing Gleason grades and tumor stages (Fig. 9b, c).

These findings align with previous studies linking *MYBL2* to high Gleason scores and poor prognosis^86^ and *RRM2* as a key driver of aggressive prostate cancer chromosomal instability of advanced-stage Pca^61,62^. *EXO1* and *NEIL3*, are both involved in DNA damage response and replication fork progression under oncogenic stress, suggesting their role in promoting genomic instability during tumor progression^63–66^. To assess the association between gene expression and PSA levels, we stratified patients into two groups (≤ 4 ng/mL, > 4 ng/mL) and applied the Wilcoxon rank-sum test (Fig. 9d). *TPX2*, *CENPA* and *RRM2* showed the strongest association with elevated PSA levels, particularly in the > 4 ng/mL group, indicative of higher tumor burden and poorer prognosis^67^. *EXO1* and *NEIL3* also exhibited moderately increased expression in PSA-high tumors, further supporting their role in disease progression. Together, these data suggest that pathways related to mitosis and DNA repair are mutually involved in PCa development and progression key genes involved in these processes might represent effective prognostic biomarkers and therapeutic targets.

**Fig. 10:**
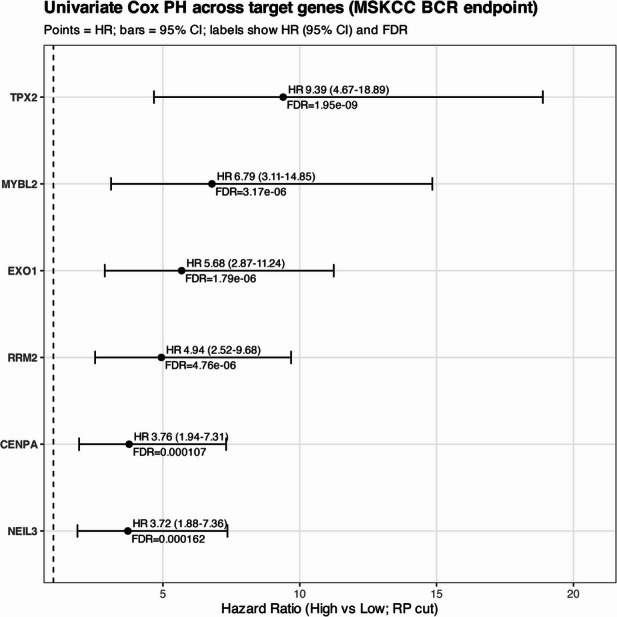
Univariate Cox proportional hazards analysis of *TPX2*-associated genes. Forest plot shows univariate Cox regression analysis of six *TPX2*-associated genes (*TPX2*, *MYBL2*, *EXO1*, *RRM2*, *CENPA*, and *NEIL3*), using biochemical recurrence (BCR) as the outcome. Hazard ratios (HR) with 95% confidence intervals (CI) are displayed for high vs. low expression groups, as defined by recursive partitioning (RP) cutpoints. After FDR correction, all six genes showed significant higher risk of BCR.

To further evaluate the prognostic impact of *TPX2* and its top associated genes, we performed univariate Cox regression analyses in the MSKCC cohort^49^. All six genes including *TPX2*, *MYBL2*, *EXO1*, *RRM2*, *CENPA*, and *NEIL3* were consistently associated with a significantly increased risk of BCR, with hazard ratios ranging from ~ 3.7 to 9.4 after FDR correction. Among these, *TPX2* emerged as the strongest predictor, with high expression conferring a 9.4-fold increased risk of recurrence (95% CI: 4.7–18.9, FDR = 1.95 × 10^–9^). These findings confirm *TPX2*’s central role and demonstrate that several of its associated genes also carry strong prognostic value for BCR in PCa (Fig. 10).

## Discussion

Understanding the molecular landscape underlying PCa progression is critical for developing targeted therapies and robust prognostic biomarkers. PCa progression is driven by complex molecular alterations that define distinct disease stages, from localized primary tumors to mHSPC towards androgen-independent mCRPC.

While risk stratification traditionally relies on clinical parameters such as Gleason score, tumor staging, and serum PSA levels, these measures alone fail to capture the molecular heterogeneity of the disease, leading to uncertainty in patient prognosis and treatment decisions^68^. Gene expression profiling has proven valuable for cancer sub-classification in different cancer entities, and several prognostic signatures or gene expression profiles determining aggressive PCa were already published or are in clinical use^69,70^.

In this study, we integrated transcriptomic profiling with ATC:hclust, WGCNA, and explainable machine learning to systematically identify core regulators of PCa progression. By leveraging a large multi-cohort dataset of 1232 patient samples spanning normal prostate, primary tumors, mHSPC, and mCRPC, we provide a high-resolution molecular landscape of transcriptional alterations across all major disease transitions and identify central biological pathways associated with mitosis, chromosome dynamics and DNA repair as driving forces of PCa initiation and progression to metastatic disease.

Based on WGCNA, *TPX2* emerged as the dominant hub gene, shared and upregulated during disease progression, underscoring its pivotal role in chromosomal instability and mitotic dysregulation, both of which are known drivers of aggressive PCa^44^. This finding distinguishes *TPX2* from previous pairwise comparisons and single-stage analyses and highlights its role as a master regulator of PCa progression^44,71^. The clinical relevance of these findings was further supported by comprehensive correlation analyses with key prognostic indicators. *TPX2* expression was significantly higher in tumors with Gleason scores ≥ 4 + 3 compared to lower-risk Gleason 3 + 3 and 3 + 4 tumors, reinforcing its role in refining intermediate-risk patient stratification^44^. A similar association of *TPX2* with high-risk disease was recently described in protein–protein interaction analyses based on transcriptomics data^72^. Additionally, *TPX2* levels progressively increased from stage T2 to T4 tumors, further associating its expression with advanced disease progression, in line with its suggested role in metastatic disease^39,73,74^. Serum PSA levels, a widely used clinical biomarker, also correlated with *TPX2* expression, with significantly higher levels observed in patients with PSA > 4 ng/mL, suggesting its potential use for identifying tumors that may require closer clinical monitoring. Intriguingly, TPX2 was previously shown to interact with the androgen receptor (AR), facilitating AR activation and upregulation of the AR target gene PSA, suggesting a direct role of TPX2 on AR signaling and disease progression^75^. Moreover, TPX2 was recently associated with the hepatoma upregulated gene (HURP) for spindle assembly and stabilisation^76^. HURP has also been described as a prognostic factor in prostate cancer associated with radiation therapy resistance^77^, thus potentially linking TPX2 networks to therapy resistance. Most importantly, TPX2 represents a central protein, not only for spindle assembly and the promotion of mitosis, but also as a regulator of DNA damage repair pathways, as recently described for pancreatic cancer^78^. In this study, targeting TPX2 was shown to provoke synthetic lethality with PARP inhibitors, which might also represent a highly valuable approach for PCa therapy.

Unlike traditional differential expression-based studies, our network-based approach together with ML-based feature selection identified not only individual biomarkers but also key regulatory interactions, uncovering novel gene co-regulation patterns that may refine prognostic models. Within the TPX2-associated network, the strongest interactions were identified between *CENPA-MYBL2* in primary localized PCa, *EXO1-NEIL2* in mHSCP and *CENPA-RRM2* in mCRPC. All these genes, together with their hub gene *TPX2* are functionally linked to mitotic control, chromosomal instability, and DNA repair pathways, key hallmarks of aggressive cancer phenotypes^45,46,79^. *CENPA* and *MYBL2* have been implicated in cell cycle regulation and transcriptional control and have been linked to aggressive tumor growth^80–82^. Moreover, a direct regulation of *CENPA* by the transcription factor MYBL2 was recently demonstrated in ovarian cancer^83^. In PCa, MYBL2 was linked to tumor cell plasticity and adaptation of tumor cells to androgen deprivation, but also to aggressive hormone-sensitive PCa^84–86^. Interestingly, MYBL2 has been implicated in the regulation of DNA repair genes including *BRCA1*, *FANC1* or *RAD51*, yet again highlighting the connection of cell cycle regulation and DNA repair pathways^87^.

While co-expression of *CENPA* and *RRM2* have been previously analyzed in breast cancer, hepatocellular carcinoma, and in murine liver regeneration, their functional relationship has not been explored in PCa so far^88–90^. RRM2 is essential for nucleotide biosynthesis and thus implicated in providing nucleotide pools for proliferation and DNA repair. In PCa, RRM2 overexpression was linked with tumor progression by promoting EMT^61^, and an association with therapy resistance, particularly docetaxel resistance via ANXA1-mediated activation of the PI3K/AKT pathway was suggested^67^. Of note, the transcription factor MYBL2 has been identified as a direct regulator of *RRM2* in colorectal cancer^91^, suggesting that a similar axis might exist in PCa. Lastly, our data highlight the rising co-expression of *EXO1* and *NEIL3* in mHSCP two additional genes implicated in DNA repair. NEIL3 has been shown to localize to replication forks during oxidative and replication stress, facilitating homologous recombination and promoting fork progression under oncogenic conditions^63,65^. EXO1 plays a complementary role, particularly in resecting stalled replication forks and resolving DNA secondary structures such as G-quadruplexes, thereby preventing genomic collapse in rapidly dividing cells^92^. Importantly, EXO1 has also been linked to aggressive PCa phenotypes, promoting lipid synthesis and accumulation, which eventually accelerated PCa progression^93^. Notably, *EXO1* levels are significantly higher in high-metastatic versus low-metastatic models, and its overexpression is associated with poor survival outcomes in primary PCa patients^94^.

Although a functional link between EXO1 and NEIL3 has not been described so far, their upregulation likely reflects a cellular adaptation to replication stress-associated DNA damage in aggressive tumors and marks a shift towards more proliferative, genomically unstable disease states^65,94^. This coordinated expression points to a potential *EXO1*–*NEIL3* axis in prostate cancer progression, warranting further investigation as a dual biomarker or therapeutic vulnerability.

In addition to 22 shared genes that were deregulated throughout PCa progression, we also identified stage-specific genes within the *TPX2* modules, such as *EZH2* and *PLK1* in mHSCP or *TERT* in mCRPC. EZH2 has been widely recognized as an essential epigenetic regulator for PCa progression, in part through direct regulation of *AR* expression^95^. Moreover, EZH2 dependent polycomb group signaling has been implicated in enzalutamide resistant prostate cancer^96^. Thus, EZH2 represents a promising therapeutic target and dual treatments targeting both the AR and EZH2 are currently undergoing clinical testing^97^. Similarly, co-treatment utilizing AR signaling inhibitors and PLK1 kinase inhibitors have been suggested to overcome PLK1 dependent chemotherapy and enzalutamide resistance in advanced PCa^98,99^. Moreover, PLK1 and TPX2 are functionally linked to control spindle pole assembly and centrosome maturation in concert with AURKA, the second centrosome kinase^100^. Finally, the upregulation of the telomerase *TERT* in mCRPC might represent an additional therapeutic vulnerability of advanced PCa. Interestingly, early prostate lesions such as prostatic intraepithelial neoplasia are associated with telomere dysfunction and rather telomere shortening^101^. Moreover, the AR directly binds the *TERT* promoter to repress its transcription in normal prostate epithelial cells, while mutations in AR result in weakened binding and *TERT* expression^102^. This finding might explain the upregulation of *TERT* specifically in mCRPC in our dataset, providing an interesting target for further evaluation.

In summary, the translational implications of our findings are underscored by the use of *TPX2* in the OncotypeDX test^52^, a clinically validated genomic panel for assessing PCa aggressiveness and guiding treatment decisions. The inclusion of *TPX2* in a commercial diagnostic assay reinforces its potential clinical utility, further validating its role as a biomarker for disease stratification. Our study extends this finding by demonstrating *TPX2*’s function not just as a standalone biomarker but as part of a larger co-regulatory network, potentially offering greater predictive power in risk assessment. These insights suggest that *TPX2*-centered biomarker signatures could be further explored for clinical applications, including prognostic modeling, therapeutic targeting, and treatment response prediction.

## Conclusion

By integrating ATC:hclust, WGCNA, and explainable machine learning, we identified key molecular drivers of PCa progression across 1232 patient samples, spanning all major disease stages. Our findings highlight TPX2 as a core regulator and provide a quantitative ranking of biomarkers with clinical potential. The identification of co-regulated gene interactions, particularly in *TPX2*-centered networks, suggests that multi-biomarker strategies may improve patient stratification and therapeutic targeting. Future studies should focus on validating *TPX2*-centered regulatory networks in prospective clinical cohorts, exploring dual biomarker combinations such as *CENPA*-*MYBL2* or *CENPA*-*RRM2*, as well as *EXO1*-*NEIL3*, and assessing their potential for informing therapeutic decision-making in precision oncology.

## Methods

### RNA-Seq data preparation

Our study is based on three combined datasets, namely the PCa Transcriptome Atlas (PCTA)^23^, the West Coast PCa Dream Team—Metastatic Castration Resistant PCa (WCDT-MCRPC)^28,29^ and GSE221601 (https://www.ncbi.nlm.nih.gov/geo/query/acc.cgi?acc=GSE221601).

PCTA is a curated collection of 11 datasets comprising PCa normal, primary and metastatic tissue RNA-Seq samples. We obtained raw expression values and metadata from the downloads section of the PCTA website (https://www.pcaprofilertest.tk). In case of replicates, we kept those sequenced using the PolyA library. If all sample replicates had identical metadata, we kept the first and discarded the rest.

WCDT-MCRPC contains 99 mCRPC RNA-Seq samples predominantly extracted from lymph nodes and bone tissues. We manually downloaded the raw STAR read counts (RNA-Seq) from the GDC data portal and the clinical annotations from dbGaP and from the NCBI SRA Run Selector website.

GSE221601 is a GEO dataset providing 52 mHSPC and 8 normal prostate tissue RNA-Seq samples. We downloaded the sra files from NCBI and manually processed them until we obtained the final STAR counts.

We combined PCTA, WCDT-MCRPC and GSE221601 into a single dataset of 1232 samples with the goal of analyzing the progression of PCa at the transcriptomic level from normal (n = 182) to primary (n = 662) to mHSPC (n = 52) to mCRPC (n = 336) samples.

### Pre-processing

While the raw counts were directly available for PCTA and WCDT samples from GSE221601 required preprocessing. We extracted the *fastq* paired-end reads from the *sra* files and used cutadapt^103^ to remove unwanted sequences (e.g., adapters, poly-A tails, etc.) and low-quality reads. Then, the STAR aligner tool^104^ was used to map the reads to the GRCh38.p13 (release 105) human reference genome, obtained from ENSEMBL (https://www.ensembl.org/Homo_sapiens/Info/Index), as well as to obtain gene counts. Only intersecting sequenced transcripts from all samples were kept, resulting in 58,210 common transcripts.

We performed three rounds of batch correction with ComBat-seq^105^ to ensure that the three datasets were as cohesive as possible and to remove any biases and sequencing artefacts. We evaluated the results of each round via PCA, and put special emphasis in preserving the biological signal given by differences in sample type. GSE221601 samples were sequenced using QuantSeq 3’, whereas samples from PCTA and WCDT were sequenced using different RNA-Seq libraries. Three rounds of batch correction were required to harmonize the data within a shared high-dimensional space, accounting for differences in library preparation and sequencing technology. We first batch-corrected the raw counts of each sample type separately in PCTA and WCDT using dataset origin and library type as batch indicators. PCTA contains samples from multiple datasets and sequenced using different library types. Next, we combined the previous result with GSE221601 samples and performed batch correction with two batches (i.e. whether the sample was inside GSE221601 or not). Finally, we repeated the first step, applying batch correction to each sample type separately, but taking also into account GSE221601 samples (Supplementary Fig. S5). The result of the third round was then combined into a single dataset again and used for downstream analyses.

### Transcriptome analysis

We used the R/Bioconductor package DESeq2^106^ to perform differential gene expression (DE) analysis of the un-stranded STAR transcript counts. We performed a naive pre-filtering step before DESeq2 to remove low count genes by only keeping those transcripts with an average count across all samples bigger than 10. Read counts were normalized by the DESeq2 normalization method of variance stabilizing transformation (VST), to be used in downstream analyses.

We initially applied the DESeq2 likelihood ratio test (LRT) to assess the overall effect of sample types on PCa progression. This approach allowed us to identify global transcriptional changes across all conditions. Then, we used pairwise comparisons for WGCNA, as it requires well-defined binary comparisons to learn discriminatory features. Differentially expressed genes (DEGs) were derived from respective experimental subgroups: primary versus normal (prim/norm), mHSPC versus primary (mHSPC/prim) and mCRPC versus mHSPC (mCRPC/mHSPC). In both sets of analysis, genes with an adjusted *P* value (Padj) < 0.05 and absolute log2 fold change (LFC) > 1 were considered significantly differentially expressed, representing a conservative and stringent approach.

### Unsupervised clustering and functional gene set enrichment analysis for differentially expressed genes

To gain insight into the biological and/or clinical relevance of the DEGs in each experimental subgroup, enrichment analysis was performed using several tools, including GO enrichment analysis, cell surface protein identification.

To define high-confidence gene expression signatures associated with distinct PCa sample types, we applied unsupervised hierarchical clustering to the top DEGs from the LRT analysis. By applying the Adaptive Two-Cluster (ATC) hierarchical clustering algorithm (ATC:hclust), we filtered out low-variance genes (sd ≤ 0.05 quantile) using the R packages cola^35^ and simplifyEnrichment^36^. The classification of samples was obtained by consensus partitioning and the signature genes were additionally clustered into two groups by k-means clustering (cluster 1, cluster 2). Furthermore, GO enrichment analysis was applied to the two groups of genes separately with the R package clusterProfiler^107^. To visualize whether the GO terms were significantly enriched in each subgroup, the binary cut was applied directly to the union of the two significant GO term lists, and a heatmap of Padj was placed on the left side of the GO similarity heatmap (Padj < 0.05). This strategy keeps all significant GO terms without removing any. The summaries of the biological functions in clusters are visualized as word clouds and are attached to the GO similarity heatmap, which gives a direct illustration of the common biological functions involved in each cluster.

### Cell surface protein analysis

We performed cell surface protein (CSP) analysis using the cell surface protein atlas (CSPA), a public resource containing experimental evidence for cell-surface proteins identified in 41 human cell types^37^. The basis and reference for the presented human surface proteome analysis was the human proteome in UniProtKB/Swiss-Prot (Version 2015_01)^108^. We matched the up- and downregulated DEGs from LRT analysis of overall effect of sample type to the human peptides from the CSPA, generating a list of cell surface proteins enriched in our data.

### WGCNA analysis

In order to obtain additional insights from our DEGs obtained from pairwise comparisons, we explored the co-expression relationships among them with the R/Bioconductor package WGCNA^38^.

Input gene expression data was pre-processed in multiple steps. First, we filtered our DEGs of interest (Padj < 0.05, abs(LFC) > 1) by keeping only those transcripts that were uniquely mapped to ENTREZID/SYMBOL and were either protein-coding or non-coding RNA. We also removed transcripts that mapped to the same ENTREZID. As a last step, we built the input matrix using the variance-stabilized transformed (VST) expression counts of our filtered DEGs.

To ensure that differences in sample size would not limit our analyses, we computed the statistical power of our pairwise comparisons using a 0.05 significance level (alpha) and effect size of 0.5 (Cohen’s d) on a two-sample *t* test, resulting in a power of 1 (prim/norm), 0.93 (mHSPC/prim) and 0.92 (mCRPC/mHSPC). These results demonstrate that our analyses were sufficiently powered to detect effect sizes of at least 0.5^109^.

We then detected modules of genes as well as their hub genes, based on node degree within each module. For this, we first performed power estimation to weight the co-expression network to approximate a scale free topology, using a signed network type and biweight midcorrelation as correlation type. More concretely, we determined the optimal power value via the scale-free topology fit index (R2) across a range of candidate powers. We selected the lowest power at which the scale-free topology fit index exceeded 0.7, ensuring that the resulting network approximated a scale-free topology.

Furthermore, we performed functional enrichment analysis for the REACTOME database^110^ using the R package clusterProfiler^107^. We compared the results from the functional enrichment analysis of each WGCNA module in each contrast to determine the most relevant modules in each comparison. Our goal was to identify the most promising module in each comparison in terms of biomarker discovery. After selecting one module for comparison, for a total of three modules, we calculated the intersection of the DEGs contained in said modules to determine the list of potential biomarkers.

### Survival analysis of TPX2-associated genes

To evaluate the prognostic relevance of the TPX2-associated genes, we performed survival analyses using the Memorial Sloan Kettering Cancer Center (MSKCC) prostate cancer cohort^49^, accessed via the prostateCancerTaylor package in R^48^. Gene expression values were log_2_ transformed and matched with clinical follow-up data on time to biochemical recurrence (BCR). For each gene, the most variable probe was selected, and patients were dichotomized into high- and low-expression groups using recursive partitioning (RP) based cutpoints. If no significant RP cutpoint was identified, the cohort was split at the median expression value. Kaplan–Meier survival curves were generated, and log-rank tests were applied to assess group differences. In addition, univariate Cox proportional hazards models were fitted to estimate hazard ratios (HR) with 95% confidence intervals (CI). *P* values from Cox regression models were corrected for multiple testing using the Benjamini–Hochberg false discovery rate (FDR).

### Generation of tissue microarrays and immunohistochemistry analysis

PCa tissues for validating immunohistochemical (IHC) staining were collected at the Department of Pathology (Medical University of Vienna, Reference No. 1413/2022). Formalin-fixed paraffin-embedded (FFPE) samples of 51 primary PCa (of which 25 were multifocal) and 35 matched lymph node metastases were utilized to generate 3 tissue microarrays (TMA) containing a total of 402 cores, including 194 primary PCa cores, 154 benign prostate gland cores, and 54 metastasis cores. TMA FFPE blocks were cut into 3 µm thick sections and manual IHC staining was performed using a Rabbit Anti-Human TPX2 antibody (11741-1-AP, Proteintech) diluted at 1:200. Counterstain was performed using hematoxylin. Interpretation of marker expression in tissue samples was performed by a pathologist trained in uropathology, using high-resolution brightfield scans performed using the Vectra Polaris™ Automated Quantitative Pathology Imaging System by Akoya Biosciences® at 40 × magnification. H-scores were calculated for each primary tumor focus, metastasis and benign prostate tissue by estimating the average % of nuclear expression in all cores of one compartment and multiplying this value by a factor based on the staining intensity (× 1 for weak staining, × 2 for moderate staining, × 3 for strong staining), resulting in a H-score ranging from 0 to 300. Estimation of an average H-score between all available cores in the final IHC slides resulted in analysis of a total of 75 primary PCa foci, 51 matched benign prostate tissues, and 32 matched metastatic tissues (Supplementary Table S9).

### Ranking of potential biomarkers with explainable machine learning

We used explainable machine learning (ML) to rank the potential biomarkers based on their ability to differentiate between sample types. More concretely, we trained a LightGBM^111^ binary classifier on these DEGs to differentiate between tissue types for each comparison (i.e. prim/norm, mHSPC/prim, mCRPC/mHSPC), and used it to calculate “importance scores” for each DEG.

We first loaded the VST expression counts and pre-processed it the same way we did for the WGCNA analysis. In addition, we scaled all VST values between 0 and 1 for better learning.

We trained the ML model following two main steps. First, we obtained the optimal hyper-parameters by applying grid search cross-validation on our data (Supplementary Table S10), as implemented in the Python package scikit-learn^112^. Second, we performed bootstrap training for 8.192 iterations. In other words, we trained the ML model multiple times, each under a different random seed, impacting the stochasticity of the training, from model fitting to train/test (80/20) data splits. For each bootstrap training iteration, we calculated the shapely additive explanations (SHAP)^113^ values for all samples, a method based on cooperative game theory used for interpretability of ML models. We calculated both individual and second-order interaction values. SHAP values were then averaged among all iterations, resulting in the final importance scores for a given contrast. It is worth noting that in the binary classification problem, the control group was set to be class 0, whereas the test group was set to be class 1 (i.e. the positive class).

Interpreting SHAP values is straight-forward. A negative or positive SHAP value for a given feature means that increased values of that feature steer the model towards a classification output of 0 or 1, respectively. The negative class (0) is the sample type used for control (e.g. norm in prim/norm) and the positive class (1) is the sample type used for the test (e.g. prim in prim/norm). Consequently, the larger the SHAP value of a feature, the bigger this feature impacts the model output.

These SHAP values supported our literature research efforts on the potential biomarkers extracted from the integrative analysis, by highlighting the most potentially interesting biomarkers, as well as possible pairwise interactions.

### Statistical analysis of clinical correlations

We used non-parametric statistical tests to determine the relationship between gene expression and clinical features (Gleason score, tumor stage, and PSA levels). Shapiro–Wilk tests and Q-Q plot inspections confirmed that gene expression values (VST-transformed) did not follow a normal distribution across clinical subgroups. The Kruskal–Wallis test^114^ was used to compare Gleason scores and tumor stages, while the Wilcoxon rank-sum test^115^ was employed for binary comparisons (e.g., PSA ≤ 4 vs. > 4 ng/mL). These non-parametric tests ensure robustness against skewed distributions and outliers in transcriptomic clinical data.

The source code used for our analysis are available in a GitHub repository for reproducibility and further research (https://github.com/CarlosUziel/pca_wgcna_ml).

## Supplementary Information

Below is the link to the electronic supplementary material.

Supplementary Material 1

Supplementary Material 2

Supplementary Material 3

Supplementary Material 4

Supplementary Material 5

Supplementary Material 6

Supplementary Material 7

Supplementary Material 8

Supplementary Material 9

Supplementary Material 10

Supplementary Material 11

## Data Availability

Data used in this study were extracted from the PCa Transcriptome Atlas (PCTA)^23^, the West Coast PCa Dream Team—Metastatic Castration Resistant PCa (WCDT-MCRPC)^28,29^ and GSE221601 (https://www.ncbi.nlm.nih.gov/geo/query/acc.cgi?acc=GSE221601), all of which were publicly available.

## Abbreviations

ATChclust: Ability to correlate to other rows: hierarchical clustering
BCR: Biochemical recurrence
CSP: Cell surface protein
CSPA: Cell surface protein atlas
dbGap: Database of genotypes and phenotypes
DDR: DNA damage repair
DEGs: Differentially expressed genes
FFPE: Formalin-fixed paraffin-embedded
GEO: Gene expression omnibus
HR: Hazard ratio
IHC: Immunohistochemical
LFC: Log2 fold change
LRT: Likelihood ratio test
ML: Machine learning
mCRPC: Metastatic castration-resistant PCa
mHSPC: Metastatic hormone-sensitive PCa
MMR: DNA mismatch repair genes
norm: Normal adjacent tissues
Padj: Adjusted *P* value
PCA: Principal component analysis
PCa: Prostate cancer
PCTA: Prostate cancer transcriptome atlas
prim: Primary PCa
PSA: Prostate specific antigen
PSMA: Prostate-specific membrane antigen
RP: Recursive partitioning
SHAP: SHapley Additive exPlanations
TCGA: The cancer genome atlas
TCGA-PRAD: Prostate adenocarcinoma dataset
TMA: Tissue microarrays
VST: Variance-stabilizing transformation
WGCNA: Weighted correlation network analysis
