# Supplementary material for: Unravelling TPX2-centered co-expression networks as key drivers of aggressive prostate cancer
Source: Sci Rep. 2025 Dec 16;15:43908. doi: 10.1038/s41598-025-27704-4 (PMC12708838; doi:10.1038/s41598-025-27704-4)
Supplement: Supplementary file 11 — Supplementary Material 11 [file 41598_2025_27704_MOESM11_ESM.pdf]

Supplementary information for

Unravelling TPX2-centered co-expression networks as key drivers of aggressive prostate cancer

Raheleh Sheibani-Tezerji[1,2,§], Carlos Uziel Pérez Malla[1,2,§], Gabriel Wasinger[2], Katarina Misura[1,2], Astrid Haase[2], Anna Malzer[1,2], Jessica Kalla[2], Loan Tran[1,2], and Gerda Egger[1,2,3*]

[1]Ludwig Boltzmann Institute Applied Diagnostics, Vienna, Austria

[2]Department of Pathology, Medical University of Vienna, Vienna, Austria

[3]Comprehensive Cancer Center, Medical University of Vienna, Vienna, Austria

[§]equal contribution

[*]Correspondence to: gerda.egger@meduniwien.ac.at

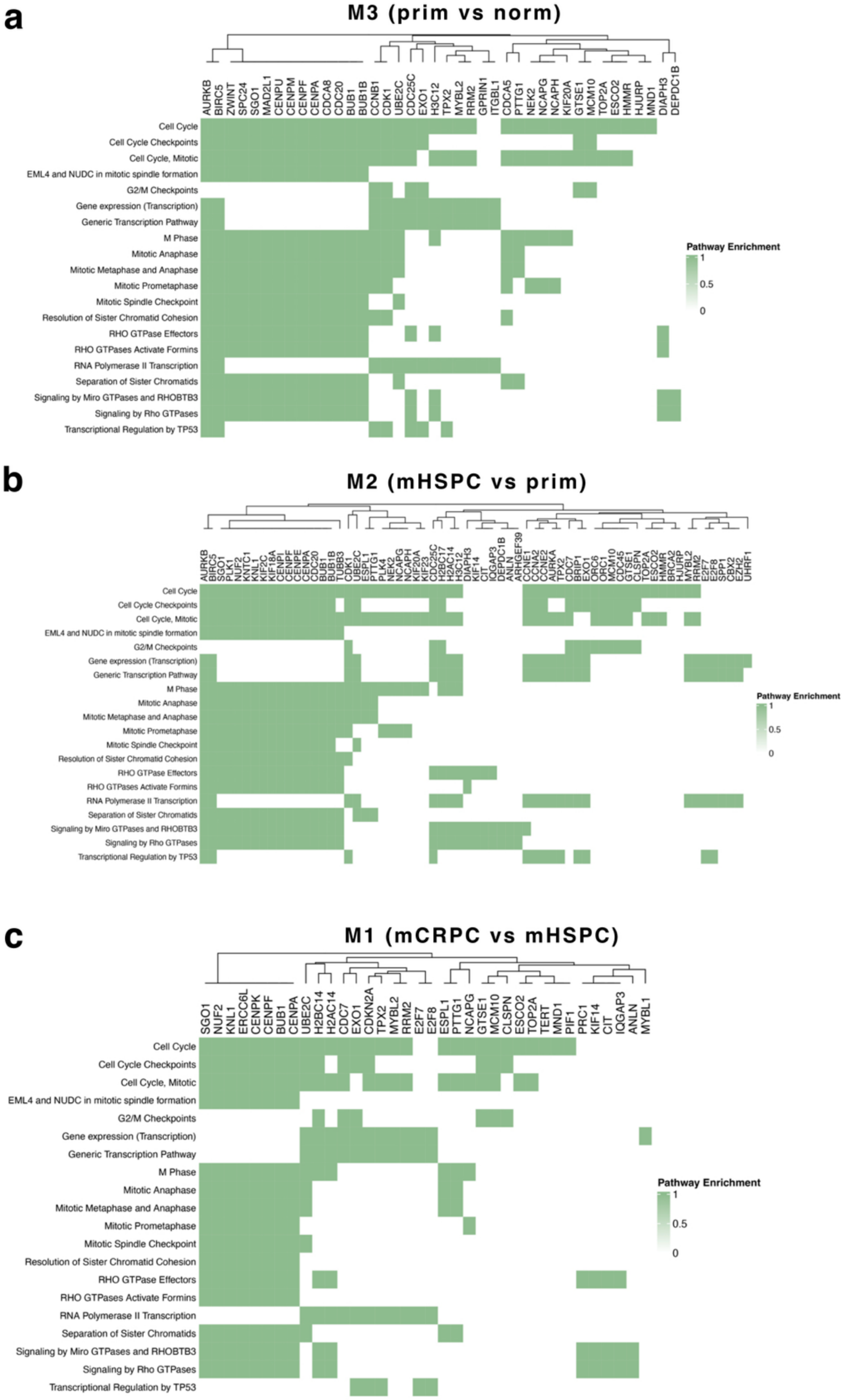

Supplementary Fig. S1. Gene content of Reactome pathways. The gene content for each shared Reactome pathways in WGCNA modules **(a)** M3 (prim/norm), **(b)** M2 (mHSPC/prim) and **(c)** M1 (mCRPC/mHSPC).

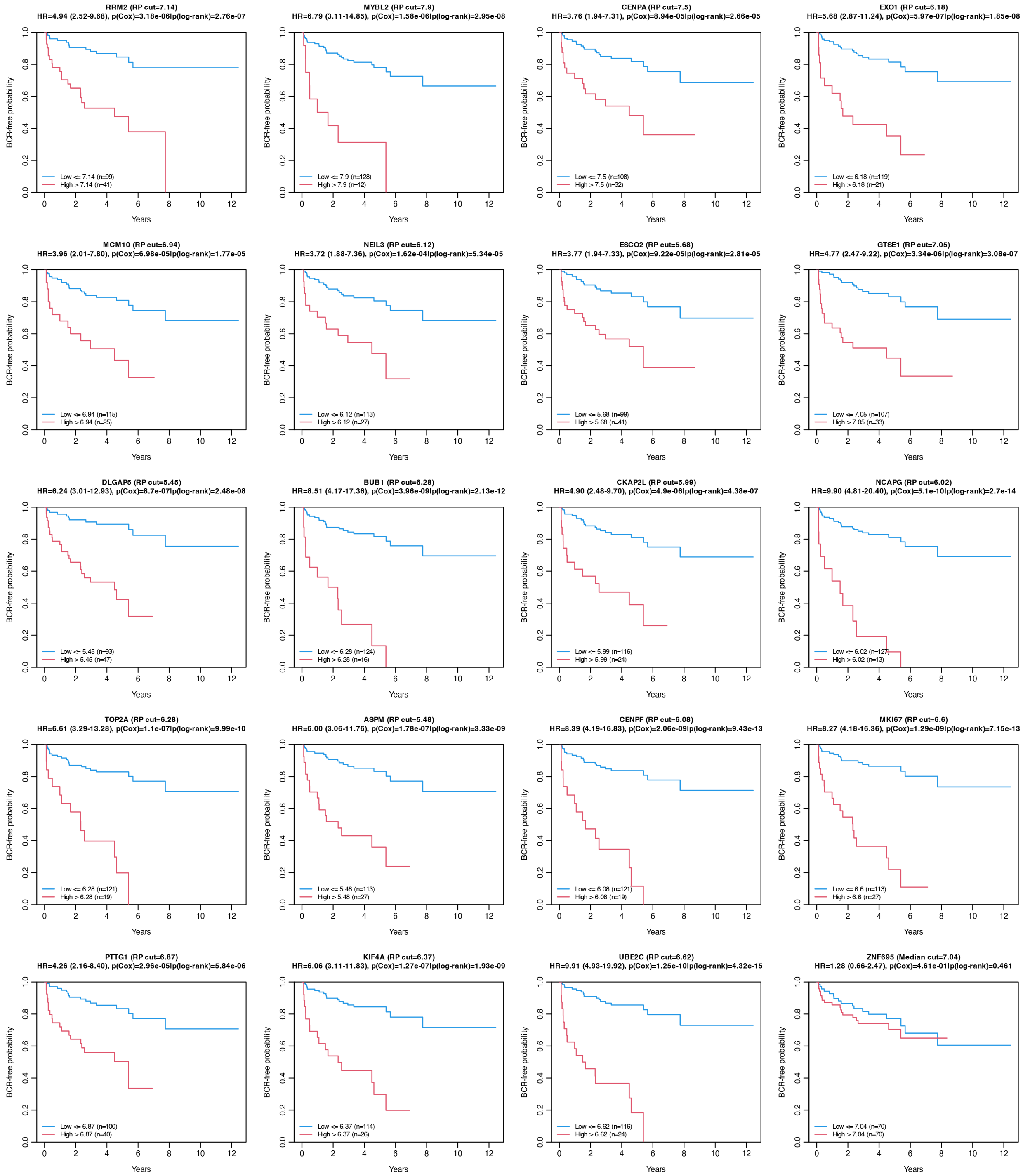

Supplementary Fig. S2. Kaplan Meyer survival curves for biochemical recurrence-free survival in the MSKCC cohort, stratified by high versus low expression of 20 out of 22 shared DEGs between M3 (prim/norm), M2 (mHSPC/prim), and M1 (mCRPC/mHSPC) comparisons. Patients were dichotomized using recursive partitioning cutpoints, except for *ZNF695* (median split). For each gene, hazard ratios (HR) with 95% confidence intervals (CI) from univariate Cox proportional hazards models, as well as log-rank p-values, are indicated in the panels.

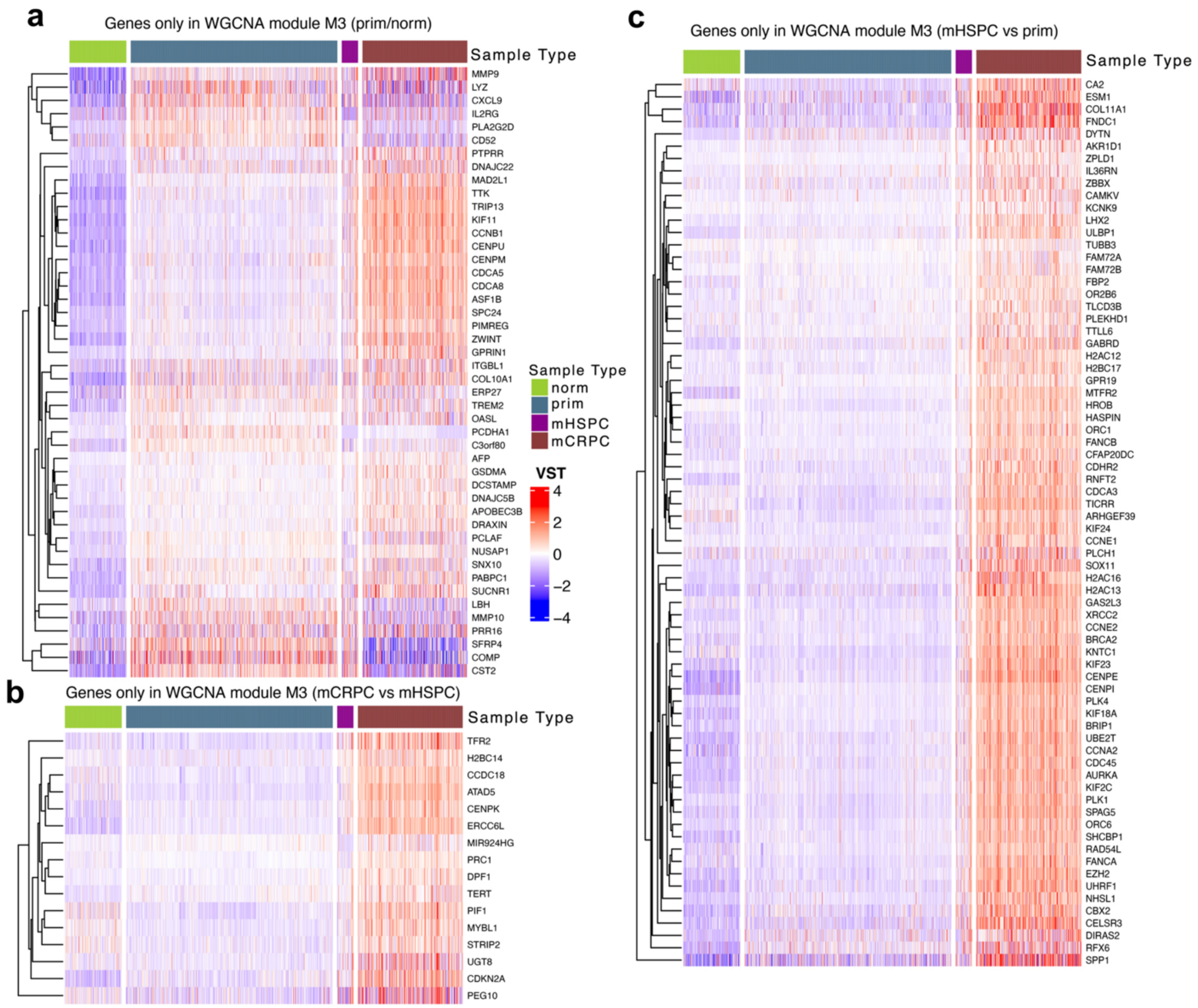

Supplementary Fig. S3. Unique genes per WGCNA module. Identified genes only present in WGCNA modules **(a)** M3 (prim/norm), **(b)** M2 (mHSPC/prim) and **(c)** M1 (mCRPC/mHSPC).

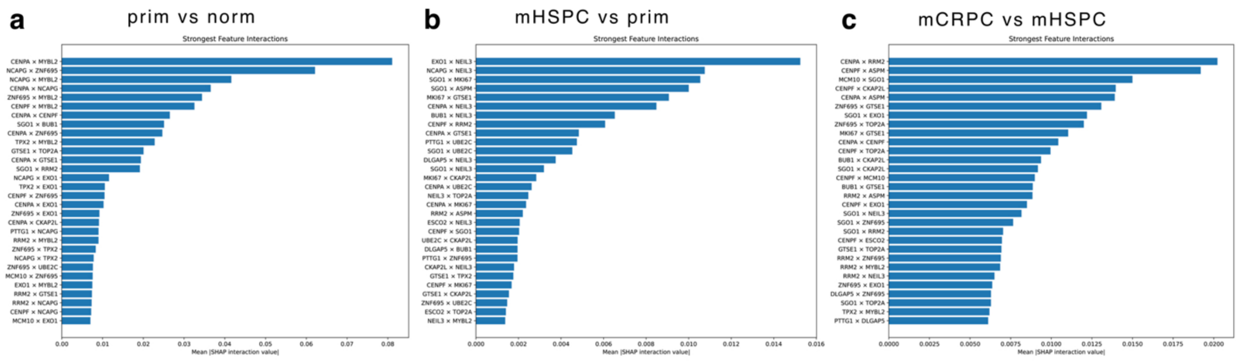

Supplementary Fig. S4. Top 30 gene pairs sorted by feature interaction SHAP values, in descending order, as obtained from binary machine learning classifiers trained on the 22 shared DEGs between **(a)** M3 (prim/norm), **(b)** M2 (mHSPC/prim) and **(c)** M1 (mCRPC/mHSPC).

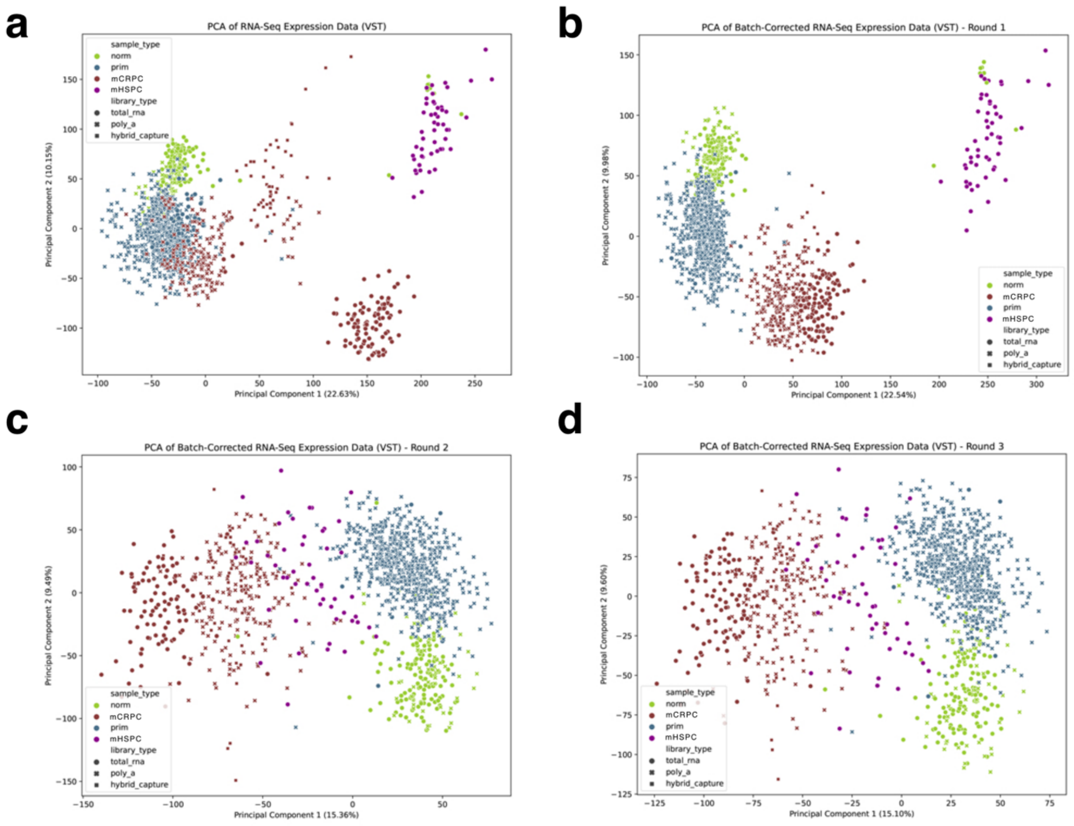

Supplementary Fig. S5. Batch correction. PCA of all samples based on VST expression values **(a)** without any batch correction, **(b)** after the first round of batch correction based on dataset origin and library type, **(c)** after the second batch correction round based on whether the sample belonged to GSE221601 or not, and **(d)** after the final batch correction round based on sample type.
